# Acquisition of complement fixing antibodies targeting *Plasmodium falciparum* merozoites in infants and their mothers in Uganda

**DOI:** 10.3389/fimmu.2023.1295543

**Published:** 2023-11-28

**Authors:** Susanne E. Mortazavi, Allan Lugaajju, Maria Nylander, Lena Danielsson, Muyideen Kolapo Tijani, James G. Beeson, Kristina E. M. Persson

**Affiliations:** ^1^ Department of Laboratory Medicine, Lund University, Lund, Sweden; ^2^ Department of Infectious Diseases, Skåne University Hospital, Lund, Sweden; ^3^ College of Health Sciences, Makerere University, Kampala, Uganda; ^4^ Clinical Chemistry and Pharmacology, Laboratory Medicine, Office for Medical Services, Region Skåne, Lund, Sweden; ^5^ Cellular Parasitology Program, Cell Biology and Genetics Unit, Department of Zoology, University of Ibadan, Ibadan, Nigeria; ^6^ The Burnet Institute, Melbourne, VIC, Australia; ^7^ Department of Infectious Diseases, University of Melbourne, Melbourne, VIC, Australia; ^8^ Central Clinical School and Department of Microbiology, Monash University, Melbourne, VIC, Australia

**Keywords:** malaria, complement, immunity, antibodies, *P. falciparum*, C1q, osteopontin, atypical b cells

## Abstract

**Background:**

Antibody-mediated complement fixation has previously been associated with protection against malaria in naturally acquired immunity. However, the process of early-life development of complement-fixing antibodies in infants, both in comparison to their respective mothers and to other immune parameters, remains less clear.

**Results:**

We measured complement-fixing antibodies in newborns and their mothers in a malaria endemic area over 5 years follow-up and found that infants’ complement-fixing antibody levels were highest at birth, decreased until six months, then increased progressively until they were similar to birth at five years. Infants with high levels at birth experienced a faster decay of complement-fixing antibodies but showed similar levels to the low response group of newborns thereafter. No difference was observed in antibody levels between infant cord blood and mothers at delivery. The same result was found when categorized into high and low response groups, indicating placental transfer of antibodies. Complement-fixing antibodies were positively correlated with total schizont-specific IgG and IgM levels in mothers and infants at several time points. At nine months, complement-fixing antibodies were negatively correlated with total B cell frequency and osteopontin concentrations in the infants, while positively correlated with atypical memory B cells and *P. falciparum*-positive atypical memory B cells.

**Conclusion:**

This study indicates that complement-fixing antibodies against *P. falciparum* merozoites are produced in the mothers and placentally-transferred, and they are acquired in infants over time during the first years of life. Understanding early life immune responses is crucial for developing a functional, long lasting malaria vaccine.

## 1 Introduction

Malaria continues to pose a significant health burden with sub-Saharan Africa bearing the major burden of disease. In 2021 there were 247 million cases and 619,000 deaths globally (1). Despite global control efforts, the reduction in malaria burden has stalled, and in the African region of the World Health Organization (WHO), it even increased during the COVID-19 pandemic (1). The majority of deaths occur in children under five years of age, and malaria in pregnancy can lead to adverse birth outcomes, including preterm delivery, low birth weight, maternal anemia, neonatal and childhood mortality and morbidity (2, 3). Moreover, there is mounting evidence that partial artemisinin resistance is increasing (1), underscoring the urgent need to develop a highly efficacious malaria vaccine.

Currently, only one malaria vaccine, RTS,S/AS01, a sporozoite protein subunit vaccine, has received WHO recommendation, but its efficacy is modest at around 55% over 12 months and 18% to 36% over 4 years, depending on age and dose regimen (4). RTS,S is now being implemented in young children 6-24 months of age. However, recent studies have shown promising results with the R21/Matrix-M vaccine, which has demonstrated an efficacy of over 80% over one malaria transmission season (5). While this level of efficacy is impressive compared to earlier trials, nearly half of the children experienced malaria within one year and immunity wanes relatively quickly over time (5, 6), indicating that there is still room for improvement of potential vaccines. In malaria-endemic regions, individuals develop naturally acquired immunity to clinical disease after repeated exposure to *P. falciparum*, and the humoral response plays a crucial role in this process (7–9). Antibodies can target various stages of the parasite life cycle, including sporozoites, blood-stage merozoites, and infected red blood cells (RBCs) (9, 10), and can reduce transmission, suppress parasite density, and control infection. Acquired immunity predominantly targets asexual blood-stage parasites to prevent diseases (11, 12), however, it is still unclear which specific antigens that mediate protective immunity and which antibody effector functions are most important.

In recent years, several studies have investigated the role of complement in antibody-mediated protection against malaria in both acquired and vaccine-induced immunity (13–18). The complement system is a critical component of innate immunity and can be activated through three pathways, namely the classical, mannose-binding lectin (MBL), and alternative pathways. In the classical antibody-dependent pathway, C1q binds to antigen-antibody complexes, which results in fixation of the complement protein C3 on target cells, ultimately leading to the formation of the membrane attack complex (MAC), which inserts into the target cell membrane and causes cell lysis. The activation of the classical complement pathway is dependent on the antibody isotype and subclass, where IgM has the highest C1q-fixing activity (12) while IgG2 and IgG4 exhibit little or no activity. Among the other IgG subclasses, IgG3 has a higher affinity for C1q than IgG1 (19, 20).

Antibody-mediated complement fixation is an important mechanism for inhibitory immune responses against various stages of the malaria parasite. Several studies have reported the involvement of complement fixation in sporozoite and merozoite lysis, which leads to the inhibition of parasite invasion and subsequent infection. For instance, sporozoite-specific IgG and IgM antibodies can fix complement on the sporozoite surface, leading to sporozoite lysis and inhibition of hepatocyte invasion (17, 21). Similarly, human merozoite-specific antibodies can cause lysis of merozoites and prevent erythrocyte invasion by activating the classical complement pathway via C1q fixation. Interestingly, C1q fixation alone can also inhibit erythrocyte invasion in the absence of other complement factors (13). Furthermore, complement-fixing antibodies against merozoite antigens have been found to confer strong protection against malaria in children living in malaria-endemic regions (13, 15). This is supported by evidence showing that high levels of IgG1 and IgG3, which can bind C1q and activate the classical complement pathway, correlate with protection from malaria (22–24).

Newborns and young infants (<3-6 months) are thought to be relatively protected from clinical malaria, but the underlying immunological mechanisms are not yet fully known. Infant immunity has traditionally been attributed to the passive transfer of maternal antimalarial IgG antibodies through the placenta in the final trimester of pregnancy (25, 26). The observed increase in malaria infections in infants coincides with the decline of maternal antimalarial IgG antibodies at 6-9 months of age (27–29), providing support for this theory. However, other factors that can influence malaria risk, such as decreased breastfeeding and the decrease in fetal hemoglobin during this period (30, 31), make it difficult to determine whether infant immunity is solely dependent on antibodies or influenced by other factors. Longitudinal studies have reported associations between maternal antimalarial antibodies and protection against infection and clinical disease in young infants (32–34). However, conflicting results have also been reported, with some studies showing no association or even an increased risk for infection, suggesting that elevated antibody levels may indicate more exposure to malaria (28, 35).

Currently, it remains unclear how the subsequent development of infant antibodies in response to natural exposure to *P. falciparum* functions to mediate protection, including the rate of their development and their antigenic targets. Furthermore, it remains uncertain whether the levels and rate of decay of infant antimalarial antibodies rely on maternal antibody levels at birth, and which specific antibodies are transferred across the placenta.

Our study aims to address this gap in knowledge by examining functional infant antimalarial antibody responses during the development of naturally acquired immunity against malaria. Specifically, we have measured the levels of anti-merozoite C1q-fixing antibodies in cord blood and compared this to the mothers’ levels to determine the transplacental ratio. We also monitored the levels in the children until they reached 5-6 years of age and explored correlations between levels of C1q-fixing antibodies and different *P. falciparum*-specific B cell subsets, as well as antibodies to *P. falciparum* schizont extract. Our study aims to provide insights into the development of functional antimalarial antibody responses in early childhood and the correlation with immune responses against malaria.

## 2 Methods

### 2.1 Study design

The first part of the study was conducted in Kasangati in Uganda, where malaria transmission is moderate (36). In brief, mother-infant pairs were enrolled at birth and mothers were sampled at birth and after 9 months. The mothers were in good health and had uncomplicated deliveries. Infants were sampled at birth (cord blood), 2.5, 6, 9 months and 5-6 years. None of the study participants exhibited fever or any signs of severe infection. During pregnancy, all women received at least one dose of Fansidar as preventive treatment and an insecticide-treated mosquito net. All provided voluntary informed consent. The study was approved by The Makerere University School of Medicine Research and Ethics Committee, The Uganda National Council of Science and Technology (approval Uganda 2007-045), and Regionala Etikprövningsnämnden in Stockholm, Sweden (2011/132-31/3).

### 2.2 Malaria diagnostics

All samples underwent testing using Rapid Diagnostic Test (RDT) pLDH/HRP2 Combo (Premier Medical Corporation Limited, India). For all positive samples, thin and thick blood smears were examined by microscopy, and parasitemia was calculated in accordance with WHO guidelines (37).

### 2.3 Parasite culture and merozoite extract preparation

3D7 *P. falciparum* was cultured at 37°C as described (38, 39) using O+ human erythrocytes at 4% hematocrit in RPMI 1640-HEPES supplemented with 1% AlbuMAX II, 25 μg/mL gentamicin, 5 mM L-glutamine and 200 μg/mL hypoxanthine. For merozoite isolation (36), synchronization of the cultures was achieved through sorbitol treatment and early to late schizont parasites were separated from uninfected RBCs by passing through MACs magnet separation columns (Miltenyi Biotec, Germany). Purified parasites were then incubated with E64 (Sigma) for 7 h, pelleted at 1900 x g 5 min, washed with PBS, resuspended in PBS, and filtered using 1.2 μm Acrodisc 32-mm syringe filters (Pall Corporation) to obtain merozoites (36). Merozoites from different preparations were pooled and homogenized through three freeze-thaw cycles. The extract contained 66 µg/ml protein (Nanodrop, ThermoFisher Scientific).

### 2.4 Complement fixation ELISA

The level of complement fixation to merozoite crude antigens was assessed as described (40) with some modifications. Maxisorp 96-well plates (Nunc, Roskilde, Denmark) were coated at 50 μL/well with frozen purified merozoites (see above) in phosphate-buffered saline (PBS) for 2 h. Between each of the following steps, the wells were washed x3 with washing buffer (PBS-0.01% Tween 20). Following 2 hours of blocking with 1% casein (ThermoFisher Scientific, Rockford, IL, USA, cat#37528), plasma samples and controls in duplicates diluted 1:50 in 0.1% casein in washing buffer were added and incubated overnight. Human C1q (10 μg/mL) (EMD Millipore Corp, USA, cat#204876) was added for 30 minutes, then rabbit anti-C1q IgG antibodies (Beeson lab) 1:2000 (41), followed by anti-rabbit IgG(H+L)-horseradish peroxidase 1:3000 (BioRad Laboratories, USA, cat#1706515), each incubation was 1 h. TMB One Solution (Promega, Madison, WI, USA, cat#G7431) was added, the reaction stopped with 1 M H_2_SO_4_ and absorbance read at 450 nm (Multiskan Sky, ThermoFisher Scientific, Rockford, IL, USA). To prevent batch/plate effects, longitudinal samples from the same mother-baby pairs were always analyzed on the same plates.

### 2.5 *P. falciparum* schizont extract ELISA

The measurement of total *P. falciparum+* IgG and IgM in plasma was conducted as previously described (42). Microtiter plates were coated with schizont extract, blocked with 5% skimmed milk (Sigma) for IgG and super block dry blend (Thermo Scientific) for IgM. Bound antibodies were quantified using TMB substrate (Promega). Absorbance was read at 450 nm.

### 2.6 Immunophenotyping of *P. falciparum* specific B cells

The immunophenotyping of *P. falciparum* B cells was conducted as previously described using flow cytometry and conjugated antibodies from BD (Becton Dickinson) (42): IgG memory B cells (CD19+CD20+CD27+FcRL4 ± IgG+), non-IgG+ memory B cells (CD19+CD20+CD27+FcRL4 ± IgG−), naïve B cells (CD19+CD20+CD27−FcRL4 ± IgG−), plasma cells/blasts (CD19+CD20−CD27+FcRL4 ± IgG−), and atypical memory B cells (CD19+CD20+CD27−FcRL4 ± IgG+). B cells specific for *P. falciparum* (Pf+) were identified using carboxyl Quantum dots (Invitrogen) conjugated to extract of trophozoite/schizont-stage parasites (43, 44).

### 2.7 Statistics

Differences in antibody levels between groups were assessed by Wilcoxon matched-pairs signed rank and by Mann-Whitney test for paired/unpaired samples, respectively. To account for repeated measurements linear mixed model was used. Continuous variables were expressed as estimated means in AR (1) covariance structure, and study participants as random effects. In two separate models changes in infants´ antibody levels over time were estimated with/without adjustment for maternal antibody levels at birth. To explore whether changes in antibody levels varied over time based on whether infants had high antibody levels (top 25% of values) at birth, an interaction term was added between time point and high value at birth. To adjust for multiple comparisons, false discovery rate was used. Analyses were performed using R Statistical Software 4.1.2 (Foundation for Statistical Computing, Vienna, Austria) and GraphPad Prism version 9.5.1 (528) for macOS (GraphPad Software, San Diego, California USA).

## 3 Results

### 3.1 Characteristics of participants

We analyzed complement-fixing antibodies in mother-infant pairs. Complement fixing activity was measured as the ability of antibodies to fix C1q, the first and essential step in the classical pathway of complement system activation. C1q fixation by antibodies to merozoites correlates with subsequent complement activation and formation of the C5-9 complex (15). We followed mother-infant pairs for 9 months from birth, including samples from mothers at birth (M0), infants at birth (B0), infants at 2.5 (B2.5), 6 (B6), and 9 (B9) months, and mothers at 9 months (M9). In addition, 39 children were sampled during a follow-up visit at 5 to 6 years of age (B5F). However, due to limitations in plasma volume, the number of samples analyzed for complement-fixing antibodies varied among the groups: M0 (n=131), B0 (n=129), B2.5 (n=95), B6 (n=104), B9 (n=112), B5F (n=39), and M9 (n=114).

### 3.2 Levels of complement-fixing antibodies in plasma in the infants

**Figure 1:**
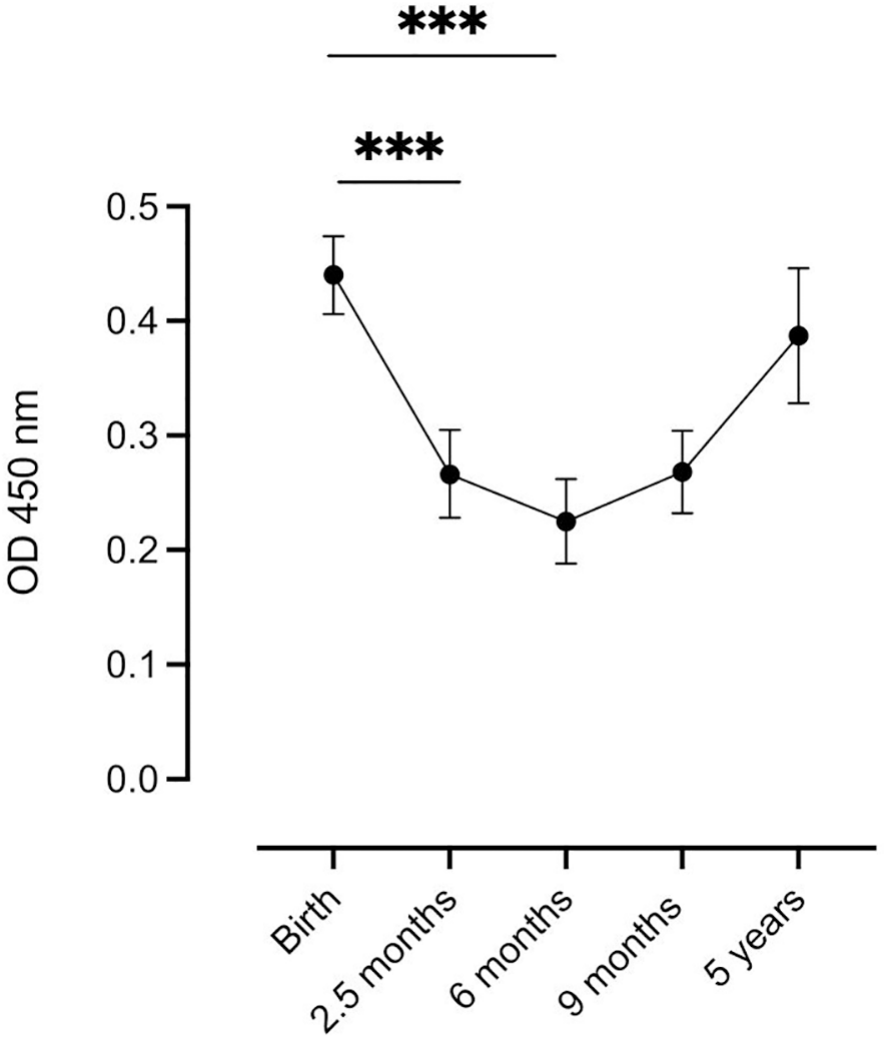
Distribution of complement-fixing antibody levels (OD 450 nm) in infants at each time point. Samples were analyzed at 5 time points: Birth (n=129), at 2.5 months (n=95), at 6 months (n=104), at 9 months (n=112), and at 5 years (n=39). Each dot represents the estimated mean (linear mixed model), and whiskers represent the 95% confidence intervals. *** indicates significance at P < 0.001.

The estimated mean levels of complement-fixing antibodies in infants at different time points were analyzed using linear mixed models, (**Figure 1**). The highest mean level was observed at birth, followed by a significant decrease in antibody levels at 2.5 months until 6 months of age. At 9 months, a slight increase in mean antibody levels was observed, but it was not statistically significant compared to the 6-month level. At 5 years of age, there was no significant difference in mean antibody levels compared to the estimated mean cord blood levels. To examine the potential influence of the maternal complement-fixing antibody levels on changes in the infant levels, the infant levels were adjusted for the maternal levels at delivery in the linear mixed model. The adjustment did not reveal any significant differences in complement-fixing antibody levels among infants at any of the five time points.

### 3.3 Levels of complement-fixing antibodies in plasma in the mothers

**Figure 2:**
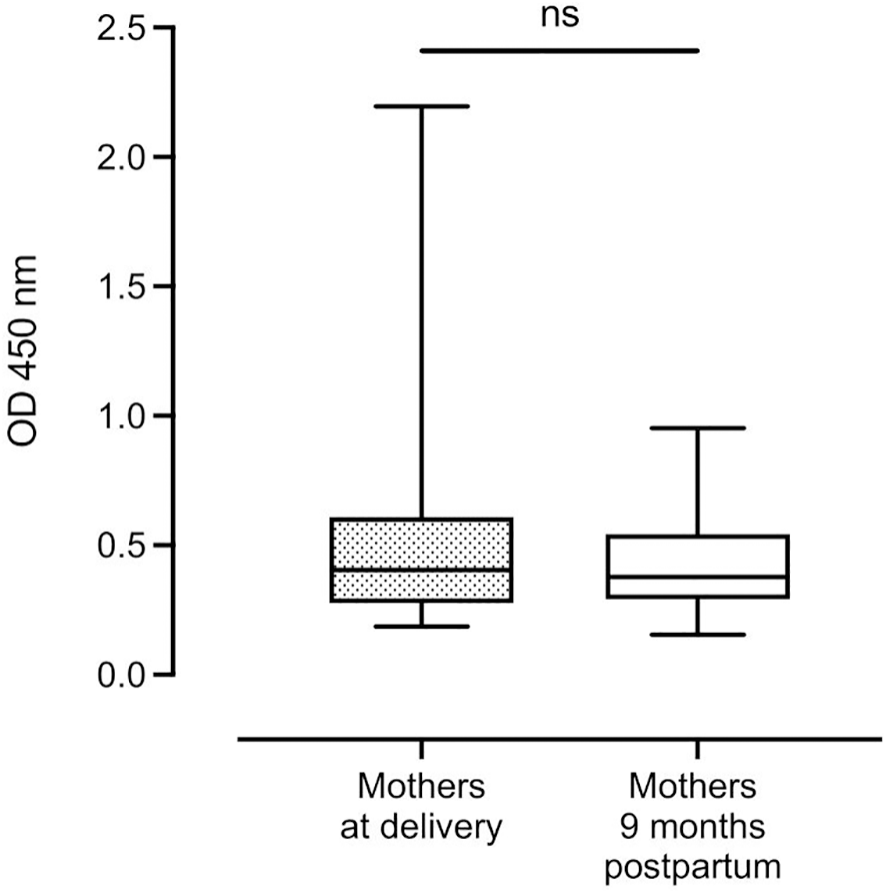
Distribution of complement-fixing antibody levels in mothers at delivery and after 9 months (n=131 and n=114, respectively). Box plots represent interquartile range, whiskers the range and horizontal lines represent the medians; ns, non-significant (Wilcoxon matched-pairs signed-rank test).

Median complement-fixing antibody levels in the mothers remained unchanged from delivery to 9 months postpartum (**Figure 2**).

### 3.4 Comparison of infants versus mothers

**Figure 3:**
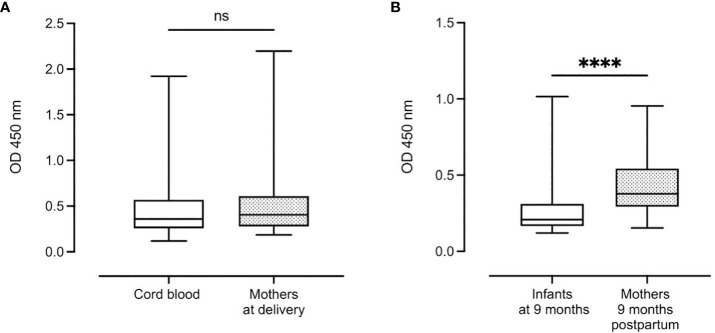
Distribution of complement-fixing antibody levels in cord blood (n=129) and mothers at delivery (n=131) **(A)** and infants and mothers after 9 months (n=112 and n=114, respectively) **(B)**. Box plots represent interquartile range, whiskers the range and horizontal lines represent the medians; ns, non-significant and ****, significant at P < 0.0001 (Mann-Whitney).

There was no significant difference in median complement-fixing antibody levels between infant cord blood (0.36 (IQR: 0.31)) and median plasma levels in mothers at delivery (0.40 (IQR: 0.33)) (**Figure 3A**). However, after 9 months, there was a significant reduction in median antibody levels in infants (0.21 (IQR: 0.14)) compared to their mothers (0.38 (IQR: 0.25), P < 0.0001) (**Figure 3B**).

### 3.5 Acquisition and decay in high and low complement-fixing antibody responders

**Figure 4:**
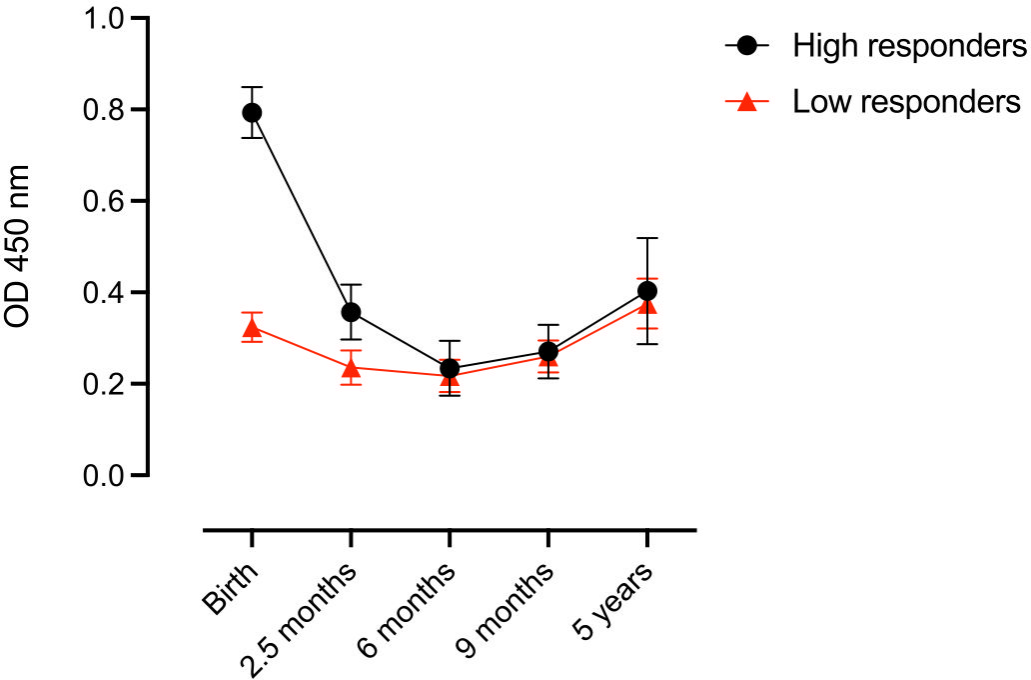
Complement-fixing antibody levels at different time points in infants categorized into high or low antibody responders at birth. Each dot represents the estimated mean (linear mixed model) and whiskers represent the 95% confidence intervals.

To assess the dynamics of complement-fixing antibody acquisition and decay, we employed a linear mixed model to compare infants based on their antibody levels at birth. Infants were categorized as high or low responders, with 25% of the highest values at birth classified as high responders (**Figure 4**). Infants with high levels of complement-fixing antibodies at birth had a faster decay of antibodies from birth to 2.5 and 6 months (estimated mean: 0.8 (CI 95%: 0.74; 0.85), 0.36 (CI 95%: 0.30; 0.42) and 0.23 (CI 95%: 0.17; 0.29), respectively) compared to those in the low response group. However, by 6 months of age, the two groups had comparable levels of antibodies and followed the same increase in levels of antibodies thereafter. When adjusting for the mothers’ complement-fixing antibody levels at delivery in the linear mixed model, we did not reveal any significant differences in antibody levels in infants at any of the five time points studied.

**Figure 5:**
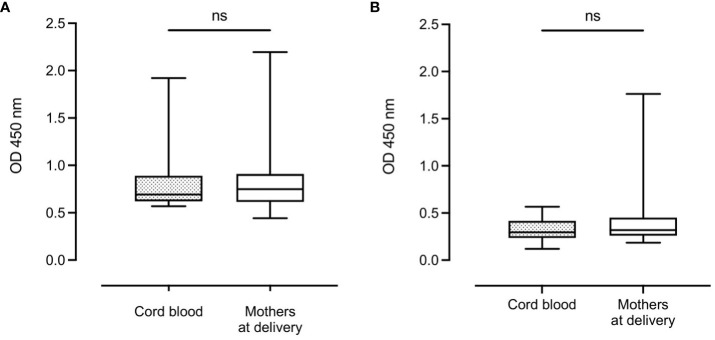
Complement-fixing antibody levels in infants and mothers at birth in the high antibody response group **(A)** and the low antibody response group **(B)**. Box plots represent interquartile range, whiskers the range and horizontal lines represent the median; ns, non-significant (Mann-Whitney test).

No significant difference in median complement-fixing antibody levels was found between infants and their mothers in the high response nor the low response group (**Figures 5A, B**).

### 3.6 Complement-fixing antibody levels and *P. falciparum* parasitemia in mothers at delivery

At delivery, among 131 mothers and 129 infants, only six mothers and one infant tested positive for *P. falciparum* using both the rapid diagnostic test (RDT) and microscopy. However, upon analyzing the complement-fixing antibody levels, we did not observe any significant differences based on the degree of parasitemia (Additional file 1). We concluded that our sample size was not large enough to detect any potential statistical differences.

### 3.7 Correlation between *P. falciparum* schizont-specific antibodies and levels of complement-fixing antibodies

**Figure 6:**
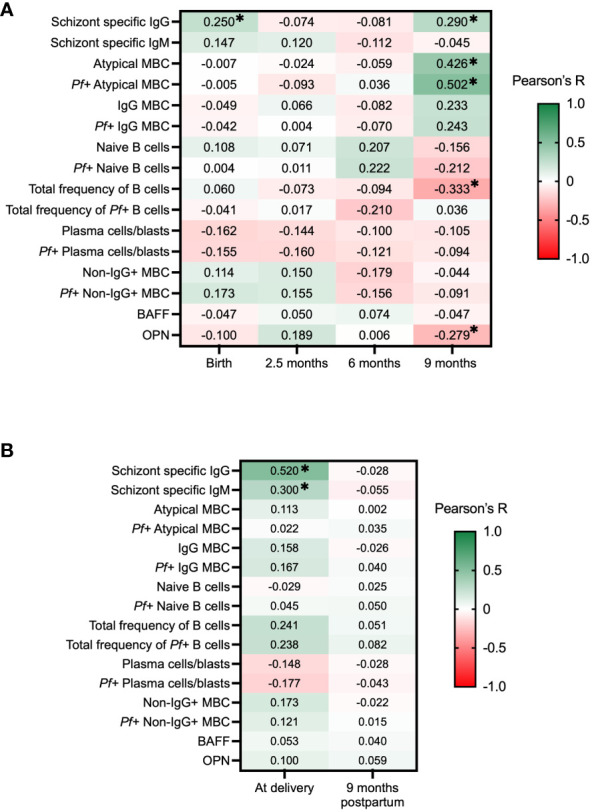
Correlation heatmap of complement-fixing antibody levels and schizont specific IgG and IgM, subsets of B cells, BAFF and OPN in infants and mothers. Pearson correlations with adjustments for multiple comparisons using the Benjamini-Hochberg method were performed for each time point, presenting R values and * represents where P <0.05. Green color represents positive correlations and red color represents negative correlations. **(A)** C1q-fixing antibody correlation heatmap for infants at birth, 2.5 months, 6 months, and 9 months. **(B)** C1q-fixing antibody correlation heatmap for mothers at delivery and after 9 months. MBC, Memory B cells; BAFF, B cell activating factor; OPN, Osteopontin.

Schizont specific IgG and IgM has been widely used as a broad marker of exposure to blood-stage malaria (42, 45, 46). During the first 9 months of follow-up, schizont specific IgG and IgM levels were analyzed in all individuals, as reported previously (42). In the current study, Pearson’s correlation was used to correlate the antibody levels with complement-fixing antibody levels, and the data was compared individually for each time point. At birth and at 9 months of age, complement-fixing antibodies were found to correlate with schizont specific IgG in the babies: r = 0.25, P = 0.02, and r = 0.29, P = 0.005, respectively (**Figure 6A**). Moreover, for mothers at delivery, complement-fixing antibody levels were found to correlate with both schizont specific IgG and IgM (r = 0.52, P < 0.0001, and r = 0.30, P = 0.01, respectively) (**Figure 6B**).

### 3.8 Correlation between total and *P. falciparum*-positive (*Pf*+) B cell subsets and complement-fixing antibodies

In order to examine the potential role of complement-fixing antibodies and B cell activities in development of immunity, we correlated our results to different proportions of B cell subsets (CD19+ cells) binding to *P. falciparum* extract (*Pf+* B cells), previously analyzed in this cohort (42). For infants, no significant correlations were seen at birth, 2.5 months, or 6 months of age. At 9 months of age, complement-fixing antibody levels were found to negatively correlate with the total frequency of B cells (r = -0.33, P = 0.005), and positively correlated with atypical memory B cells (MBCs) (defined as CD19+CD20+CD27−FcRL4 ± IgG+) (r = 0.43, P < 0.001) and *Pf+* atypical MBCs (r = 0.50, P < 0.001) (**Figure 6A**). No significant correlations were found between complement-fixing antibodies and B cell subsets in mothers at delivery or after 9 months.

### 3.9 Correlations between plasma concentrations of BAFF, OPN and levels of complement-fixing antibodies

Both OPN and BAFF have important roles in the production of immunoglobulins and B cell differentiation. Our prior studies within the same cohort have shown interesting correlations between these factors in the context of malaria (39, 47). Results showed that levels of complement-fixing antibodies in infants were negatively correlated with OPN concentrations at 9 months of age (r = -0.28, P = 0.048) (**Figure 6A**).

## 4 Discussion

Understanding the functional mechanisms that underlie naturally acquired immunity against malaria is of critical importance for the development of an effective vaccine. However, the exact process by which immunity is acquired is not yet fully understood especially among young children who are the primary target of malaria vaccines. In this study, we present, for the first time, the dynamics of complement-fixing antibodies in a longitudinal cohort of healthy mother-infant pairs residing in a malaria-endemic region of Uganda, from birth until five years of age. We show that complement-fixing antibodies in infants gradually decrease during the first six months of life, probably due to natural decay of antibodies transferred from the mothers. We further demonstrate that by five years of age, the antibody responses have nearly reached the same levels as those observed in both cord blood at birth and in the mothers.

In malaria-endemic regions, it is widely acknowledged that children develop partial immunity against malaria during their first few years of life, while infants below 4-6 months of age seldom experience clinical malaria, despite the possibility of having sub-microscopic and asymptomatic infections (48–51). This is partly due to the transfer of maternal antibodies through the placenta, which facilitates a more rapid spontaneous clearance of parasites in young infants when compared to older infants and children (52, 53). The transfer of antibodies through the placenta is predominantly restricted to the IgG subclasses, and among them, IgG1 demonstrates the highest efficiency in antibody transfer (54). In general, maternal and fetal IgG concentrations become comparable by 33 weeks of gestation (55). Towards the end of the third trimester, fetal IgG levels can even exceed those of the mother (56–59). However, studies conducted in African settings have reported lower cord/maternal IgG ratios and reduced total IgG transfer across the placenta (58, 60), indicating impaired transfer. This phenomenon can be attributed to various factors, including HIV infection (61), placental malaria (62, 63) and elevated maternal IgG levels, which are common in areas with continuous pathogen exposure (58, 60, 63). Maternal IgG antibodies are likely to play a significant role in the presence of complement-fixing antibodies detected in cord blood, as fetal-produced IgG represents only a small fraction of the immunoglobulins at birth (64). In our study, we found very similar levels of complement-fixing antibodies in both cord blood and in mothers at delivery, even when categorized into high and low responders. These findings strongly indicate the transfer of complement-dependent antibodies across the placenta, without any compromise observed in the transmission of complement-fixing IgG to the infants within our cohort. IgM responses were seen among mothers, and IgM can effectively fix and activate complement. However, IgM is not transferred across the placenta.

When we correlated our data for complement-fixing antibodies to schizont-specific IgG and IgM, we were surprised to discover correlations in the mothers only at delivery, but not nine months later. The majority of the mothers have resided in the same area for several years, encountering malaria multiple times, suggesting a relatively long-developed immunity. Interestingly, a previous study on individuals with clinical malaria reported strong associations between complement-fixing antibodies and merozoite-specific IgM as well as subclasses IgG1 and IgG3 (40). Our results could suggest a more recent exposure to malaria for the mothers at the time of delivery, compared to nine months later. Previous research on pregnant women with malaria exposure has also reported correlations between complement-binding antibodies and IgM and IgG responses, mainly consisting of IgG1 and IgG3 subclasses, which are known to have a high affinity for complement (18, 65). It is worth noting that high levels of complement-fixing antibodies during pregnancy have been linked to a lower risk of placental parasitemia at delivery, implying a more effective control of the infection (65). However, in our study, we did not observe such an association due to very few mothers having enough parasites in the blood to be measured by our methods. Among the 131 participating mothers, six were found to have confirmed parasitemia at delivery through RDT and microscopy. Interestingly, the levels of C1q-fixing antibodies varied widely among these six women, ranging from low to high values, with two of them belonging to the high antibody response group. Nevertheless, it is challenging to draw any definitive conclusions regarding the potential protective immunity for these mothers. A limitation of our study is that evaluation of *P. falciparum* parasitemia using more sensitive molecular methods, such as qPCR, was not performed; therefore, we may have missed infections. None of the participants in our cohort presented with malaria symptoms and those with parasitemia were most likely asymptomatic carriers of *P. falciparum*, or at least did not have fever or other obvious symptoms of infection. All 109 women had received intermittent preventive therapy and mosquito bed nets during their pregnancies, which could explain the low prevalence of parasitemia. Given that all women resided in close proximity to the clinic and needed to bring their children for multiple follow-up visits, we anticipate that the transmission of malaria would be similar among all individuals.

In our assay, merozoite extract is bound to the plate and both IgG and IgM can bind to the merozoites, even though we only measure the levels of IgG. This could also mean that if there are very high levels of IgM, it could compete with binding of IgG. Nevertheless, considering the decline in IgG levels among infants in the months following birth, we do not consider this to be a concern for the overall interpretation of our results, even though it might have the potential to influence certain individual outcomes.

Maternal antibodies that develop during pregnancy have been shown to play a role in improving birth outcomes for the babies. However, the mechanisms underlying their protective effects remain poorly understood (66). It has been suggested that complement-binding antibodies may contribute to the development of immunity against pathogens (65). Our study found a correlation between schizont-specific IgG antibodies and complement -fixing antibodies in cord blood, indicating the existence of transfer of functional malaria-specific antibodies in parallel to other *P. falciparum*-specific IgG from mothers to infants, providing early-life protection. Our findings align with previous studies on malaria-specific antibody responses in sub-Saharan African birth cohorts (29, 51, 59, 67), as we observed a decline in complement-fixing antibody levels in infants during the initial six months, followed by an increase at nine months. This pattern reflects the waning of maternal antibodies and the infant’s own acquisition of antibodies.

At nine months of age, we found several significant correlations in the infants when compared to other immune response parameters. Specifically, we observed that complement-fixing antibodies were positively associated with schizont-specific IgG, atypical memory B cells (MBCs), and *Pf+* atypical MBCs, all of which exhibit an increase in response to malaria exposure. Although previous studies have shown correlations between the acquisition of atypical MBCs, exposure to malaria, and age, the role of these cells in the formation of immune responses against malaria remains unclear (39, 42, 68, 69). Moreover, our findings indicated a correlation between complement-fixing antibodies and OPN at 9 months, suggesting a potential connection between complement-fixing antibodies and the regulation of B cell differentiation through OPN-mediated pathways. Both OPN and BAFF might have a role in the B cell immune response against *P. falciparum* infection and the formation of atypical MBCs (39, 47), but its precise function in this context is not yet fully understood. We speculate that the infant’s immune system becomes highly active in developing its own immunity against malaria around the age of 9 months. This heightened activity may explain the presence of multiple correlations, such as those observed with *P. falciparum*-specific B cells at this time point. In contrast, such correlations may be less pronounced during other ages when the immune system has reached a more stable state. Upon reaching the age of 5 years in our study cohort, the levels of complement-fixing antibodies in children appear to be comparable to those of their mothers. This is consistent with findings from a study conducted in a malaria-endemic region of Kenya, which reported that children at the age of 5 years had significantly higher C1q-fixing antibody levels against the merozoite surface protein 2 (MSP2) compared to younger children, and had reached similar levels to those observed in adults (16). However, in the same study, complement-fixing antibody levels against the circumsporozoite protein (CSP) were lower in children under 5 years old compared to adults (16). This indicates that the development of complement-fixing antibodies against sporozoite antigens may be slower than that of merozoite antigens. Nonetheless, our results likely reflect the gradual development of protective immunity against clinical malaria during childhood. This is also supported by previous studies that demonstrated levels of complement-binding antibodies against CSP and a number of merozoite antigens to be associated with protection from clinical malaria in children (13, 15, 16). Therefore, our findings suggest that by the age of 5 years, children have developed functional complement-binding antibodies that may contribute to protection against malaria.

Upon dividing the infants into high and low complement-fixing antibody responders at birth, our findings revealed a faster decay rate of maternal complement-fixing antibodies in infants with initially high antibody levels. These results are in line with a study conducted on a birth cohort in Kenya, which demonstrated that the rate of decline of maternal total IgG levels against five recombinant merozoite antigens was inversely proportional to the starting levels in cord blood (70). Moreover, it has been demonstrated that maternal antibodies against viral agents such as rubella (71), parainfluenza type 3, and influenza A2 (72) decline more rapidly in children with high levels of cord blood antibodies. The inverse relationship between starting levels and decay rate has been attributed to an increase in the rate of catabolism due to increased serum IgG levels (71), or an increased antibody consumption secondary to asymptomatic malaria infections (70). The rate of decline of maternal IgG can also vary depending on the antigen-specificity, as antibodies against MSP2 have been shown to have a shorter half-life compared to AMA1, for example (73). Notably, at 6 months of age the high and low level groups were equivalent in our study, indicating that the levels of complement-fixing antibodies in cord blood and in the mothers do not influence the production of the infants’ own antibodies. These findings, together with the fact that the mothers’ complement-fixing antibody levels were consistent with those of their respective infants in both groups, confirm that complement-fixing antibodies are transferred via the placenta and that the levels in cord blood are primarily dependent on maternal levels during pregnancy.

The observed variations in complement-fixing antibody levels between the different mother-infant pairs in our cohort may be attributed to differences in exposure to *P. falciparum*, as evidenced also by prior studies indicating that high levels of exposure result in high levels of antibodies (51, 74). High IgG antibody levels in cord blood have been reported as an indicator of frequent malaria exposure during gestation and have been found to correlate with an increased risk of malaria infection during the first year of life (51, 74). Notably, while high MSP1-antibody levels in cord blood have been associated with protection of infants from malaria infections in high-endemicity settings during the first 6 months of life, they have also been associated with a higher risk of infection compared to infants in low-endemicity settings (75). Considering these observations, it would be interesting to investigate whether infants with higher levels of maternally derived complement-fixing antibodies experience longer protection from malaria infections and a shorter time-to-first-infection following the waning of maternal antibodies, compared to those with lower levels.

In conclusion, this study provides novel insights into the longitudinal decay and acquisition of complement-fixing antibodies in a birth-cohort residing in a malaria-endemic region, and it also clearly indicates that these antibodies are transferred effectively across the placental barrier. However, further studies are necessary to assess functional antibodies and neonatal, maternal, and fetal factors that lead to clinical immunity. Understanding the development of the immune response in infancy and early childhood is crucial since children under 5 years are the primary target population for current and future malaria vaccines.

## Data availability statement

The original contributions presented in the study are included in the article/**Supplementary Material**. Further inquiries can be directed to the corresponding author.

## Ethics statement

The studies involving humans were approved by The Makerere University School of Medicine Research and Ethics Committee, The Uganda National Council of Science and Technology (approval 2011-114), and Regionala Etikprövningsnämnden in Stockholm, Sweden (2014/478-32).

## Author contributions

SM: Conceptualization, Investigation, Methodology, Validation, Visualization, Writing – review & editing, Data curation, Formal Analysis, Writing – original draft. AL: Data curation, Writing – review & editing. MN: Data curation, Formal Analysis, Investigation, Writing – review & editing. LD: Data curation, Formal Analysis, Methodology, Writing – review & editing. MT: Conceptualization, Investigation, Methodology, Writing – review & editing. JB: Conceptualization, Investigation, Methodology, Writing – review & editing. KP: Conceptualization, Investigation, Methodology, Writing – review & editing, Funding acquisition, Project administration, Resources, Supervision, Validation, Visualization, Formal Analysis.
