# Supplementary material for: Acquisition of complement fixing antibodies targeting Plasmodium falciparum merozoites in infants and their mothers in Uganda
Source: Front Immunol. 2023 Nov 28;14:1295543. doi: 10.3389/fimmu.2023.1295543 (PMC10715273; doi:10.3389/fimmu.2023.1295543)
Supplement: Supplementary file 1 [file Table_1.docx]

Supplementary Material

# Supplementary Tables

| **C1q-fixing antibody level (OD)** | \| **Parasitemia number of infected RBC/µL of blood** \| \| --- \| |
| --- | --- | --- |
| 0.32 | 48 |
| 0.82 | 16 |
| 0.61 | 240 |
| 2.2 | 64 |
| 0.87 | 32 |
| 0.36 | 2760 |

**Supplementary table 1.** C1q-fixing antibody levels and parasitemia in mothers at delivery

| **C1q-fixing antibody level (OD)** | \| **Parasitemia number of infected RBC/µL of blood** \| \| --- \| |
| --- | --- | --- |
| 0.62 | 13 000 |

**Supplementary table 2.** C1q-fixing antibody levels and parasitemia in infants at delivery
